# Value of extracorporeal artificial liver support in pediatric acute liver failure: A single-center experience of over 10 years

**DOI:** 10.3389/fped.2023.979619

**Published:** 2023-02-13

**Authors:** Ilhan Ocak

**Affiliations:** Department of Liver Transplant Intensive Care Unit, Memorial Sisli Hospital, Şişli, Turkey

**Keywords:** acute liver failure (ALF), continuous renal replacement therapy (CRRT), hepatic encephalopathy (HE), liver transplantation (LTX), plasma exchange (PEX)

## Abstract

**Purpose:**

Acute liver failure (ALF) is a life-threatening disease characterized by rapid-onset liver dysfunction, coagulopathy, and encephalopathy in patients without chronic liver disease. Today, the combined application of continuous veno-venous hemodiafiltration (CVVHDF) and plasma exchange (PEX), which are forms of supportive extracorporeal therapy (SECT), with conventional liver therapy in ALF is recommended. This study aims to retrospectively analyze the effects of combined SECT in pediatric patients with ALF.

**Materials and Methods:**

We retrospectively analyzed 42 pediatric patients, followed in the liver transplantation intensive care unit. The patients had ALF and received PEX supportive therapy with combined CVVHDF. The biochemical lab values of the results for the patients before the first combined SECT and after the last combined SECT were analyzed comparatively.

**Results:**

Of the pediatric patients included in our study, 20 were girls and 22 were boys. Liver transplantation was performed in 22 patients, and 20 patients recovered without transplantation. After the discontinuation of combined SECT, all patients had significantly lower serum liver function test results (total bilirubin, alanine transaminase, aspartate transaminase), ammonia, and prothrombin time/international normalized ratio levels than the previous levels (*p* < 0.01). Hemodynamic parameters (i.e., mean arterial pressure) also improved significantly.

**Discussion and Conclusion:**

Combined CVVHDF and PEX treatment significantly improved biochemical parameters and clinical findings, including encephalopathy, in pediatric patients with ALF. PEX therapy combined with CVVHDF is a proper supportive therapy for bridging or recovery.

## Introduction

Acute liver failure (ALF) is a life-threatening disease characterized by rapid-onset liver dysfunction, coagulopathy, and encephalopathy in patients without chronic liver disease. Acute-on-chronic liver failure (ACLF) is used to describe the occurrence of ALF with sudden and life-threatening worsening of clinical conditions in patients with cirrhosis or chronic liver disease. ALF has a mortality rate of up to 90% and is higher in patients younger than 12 months of age (1, 2). In the pathophysiology of the disease, the hypothesis of endogenous intoxication is accepted. Endogenous intoxication causes several metabolic disorders, including hepatic coma and multiorgan failure (MOF) (3–6). Disseminated intravascular coagulation (DIC), acute renal failure (ARF), shock due to the loss of vascular resistance, acute respiratory distress syndrome (ARDS), hepatic encephalopathy (HE), and sepsis-associated infection translocation often develop secondary to ALF. The severity of organ failure is linked to elevated and circulating cytokines and endotoxins, such as TNF-alpha, interleukin (IL)-6, IL-1ß, IL-8, IL-10, IL-2, IL-4, and IFN-*γ* (7–9). Although some patients with a high probability of death may recover with supportive treatment, emergency liver transplantation (LTX) still is the definitive treatment; however, the availability of suitable organs for LTX is limited. A combination of continuous veno-venous hemodiafiltration (CVVHDF) and plasma exchange (PEX) is applied as a bridging treatment both in recovery without LTX and to prolong treatment until LTX (10). This study aims to retrospectively analyze the effects of combined supportive extracorporeal therapy (SECT) in pediatric patients with ALF.

## Technical aspects

### Plasma exchange

PEX can be performed by centrifugation or filtration-based mechanisms. Centrifugation separates the blood into its components by density (specific gravity), while in filtration, components of the blood are separated according to their size by passing through a perforated membrane, according to the pore diameter of the membrane. Centrifugation and filtration-based systems are similar in terms of safety, efficiency, and therapeutic effect. As the shaped cells of the blood are returned to the patient, the plasma is eliminated and replaced with fresh-frozen plasma (FFP), albumin, or another liquid. The use of FFP allows for the replacement of important and highly beneficial substances, such as albumin and coagulation factors, in addition to substances that act on mediators of inflammation. Typical PEX therapy replaces 1–1.5 times the patient's estimated plasma volume. Plasma volume is calculated from the estimated total blood volume using common physiological variables, such as an individual's sex, height, weight, body muscle mass, and hematocrit. An exchange of 1–1.5 times the plasma volume removes approximately 70% of the substances from the intravascular space.

### Continuous veno-venous hemodiafiltration

CVVHDF combines two continuous renal replacement therapies (CRRTs): hemodialysis and hemofiltration. It can clear both low- and medium-to-high-molecular-weight molecules by combining diffusion and convective methods. Diffusion is the movement of a solute along a semipermeable membrane according to a concentration gradient. The clearance depends on the concentration gradient, the size of the solute, protein binding, and the surface area of the filter. Diffusion eases the clearance of low-molecular-weight solutes, such as urea and creatinine. Convection involves the movement of water and dissolved solutes across a semipermeable membrane due to a pressure gradient. Depending on the membrane porosity, medium-to-large molecules, such as cytokines, can be scavenged by convection.

### Materials and methods

The data of 424 pediatric patients who underwent LTX and 48 patients who followed up without transplantation were retrospectively analyzed. The data were collected from the intensive care unit (ICU) of Memorial Sisli Hospital, Istanbul, Turkey, where an average of 100 LTXs per year were performed between January 2010 and January 2020. In this ICU, pre- and post-transplant support and treatment were performed.

**Figure 1:**
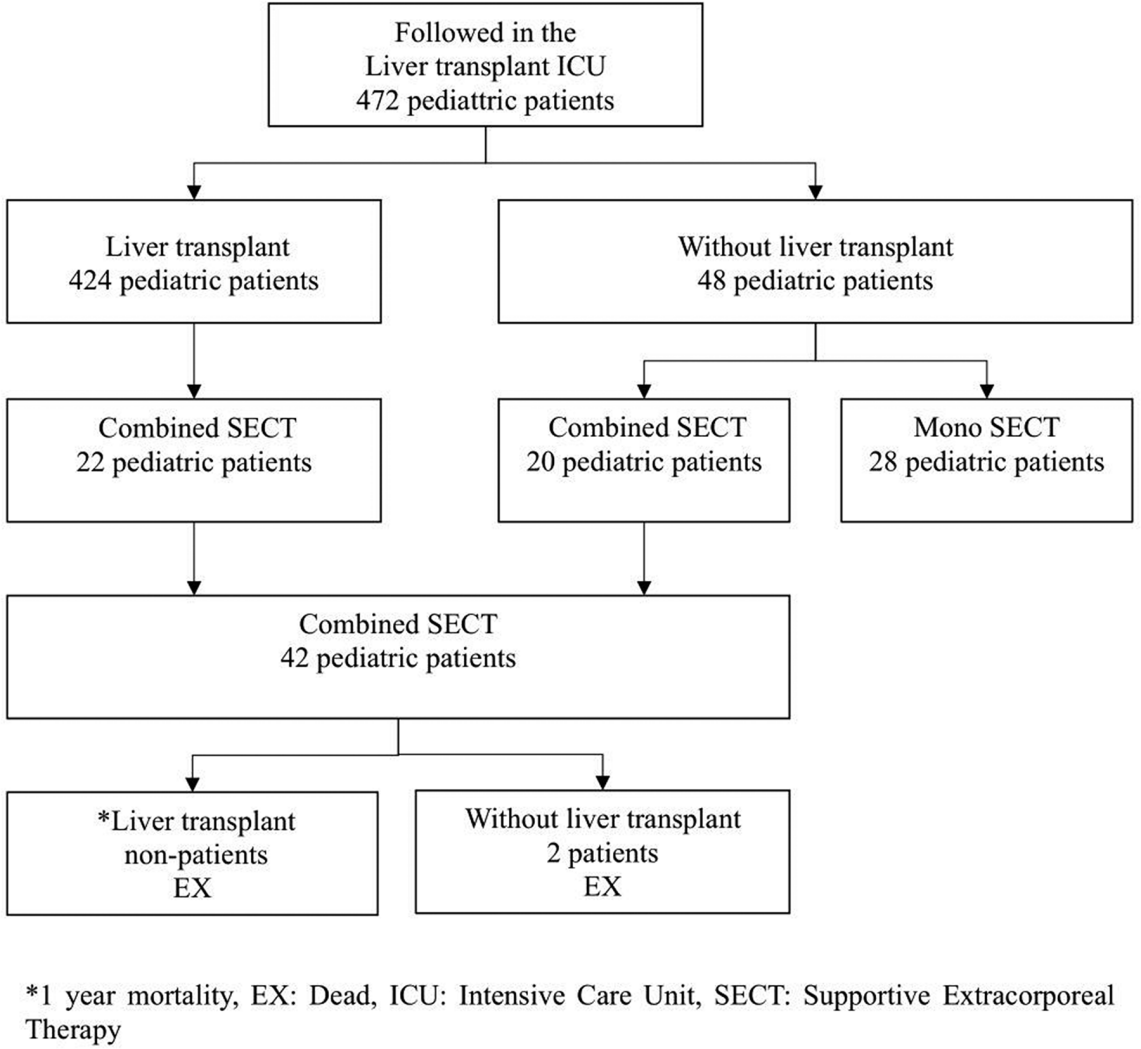
Flow diagram of pediatric patients in the study.

**Figure 2:**
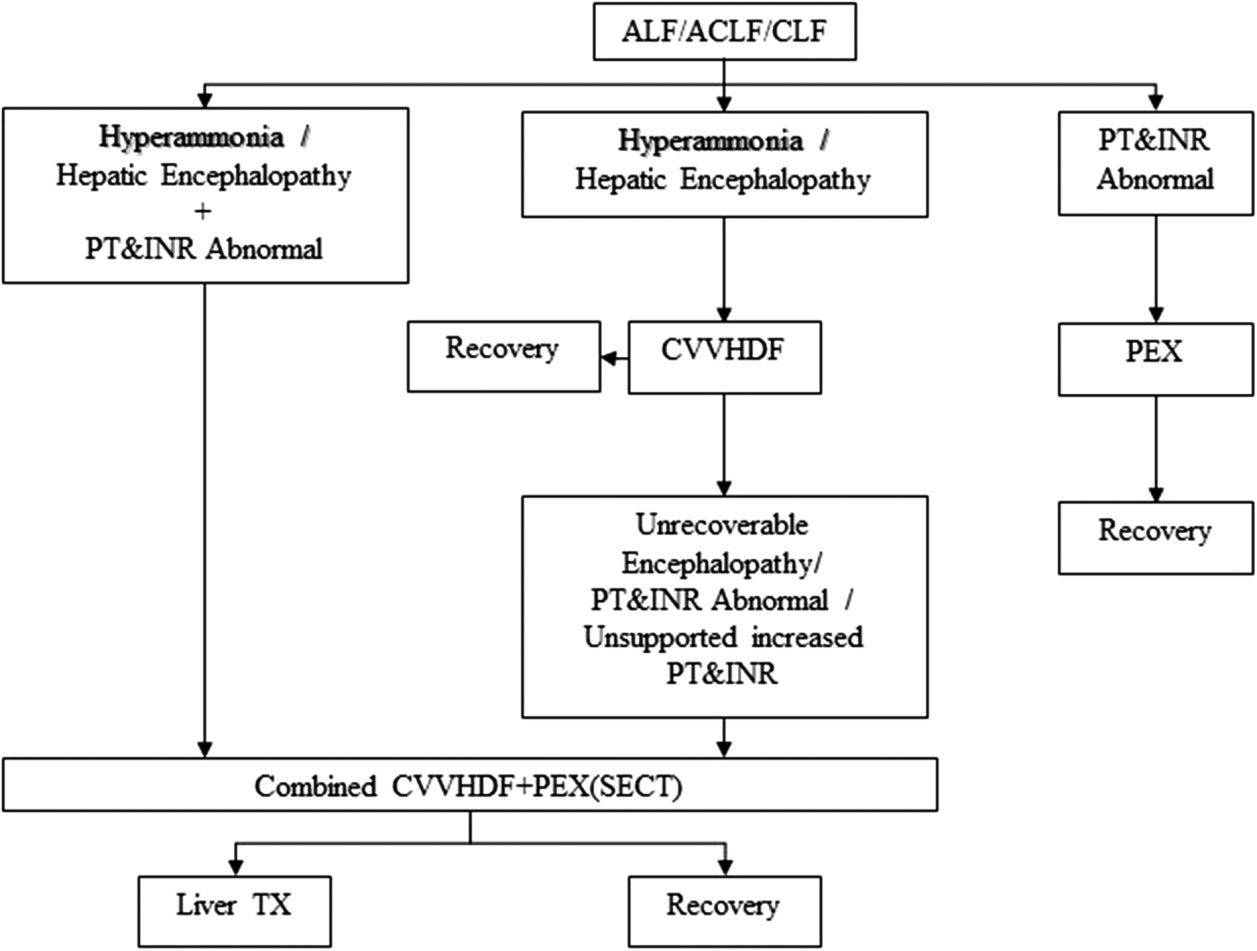
ECT protocol for ALF, ACLF, or CLF. ECT, extracorporeal therapy; ALF, acute liver failure; ACLF, acute-on-chronic liver failure; CVVHDF, continuous veno-venous hemodiafiltration; CLF, chronic liver failure; PEX, plasma exchange; PT, prothrombin time; INR, international normalized ratio; Liver TX, liver transplantation; SECT, supportive extracorporeal therapy.

Twenty ALF pediatric patients and 22 ALF developing liver transplant recipient candidates who received a combination of CVVHDF and PEX supportive therapy were included in the study. The group consisting of 22 patients was a candidate for liver transplantation according to different etiology and the Pediatric End-Stage Liver Disease (PELD) score and was on the organ transplantation waiting list. This group, which was CLF, converted to ALF for various reasons during the waiting period for transplant. In other words, it was ACLF. The living donor of this group was ready or being prepared and was on the waiting list. The other group consisting of 20 pediatric patients developed ALF due to different etiologies. The inclusion criteria of these two groups were combined SECT (CVVHDF + PEX) until recovery or liver transplant. Patients with ALF who did not undergo combined SECT or who only underwent mono SECT were excluded from the study (Figure 1, Table 1). A SECT protocol used in the ICU was applied (Figure 2) (11).

**Table 1:** Data of pediatric patients at the time of admission to the transplant ICU.

Followed in the LTX intensive care unit	472
Included patients	
Combined SECT	42
Excluded patients	
Liver transplant without combined SECT	422
Mono SECT	28
**Combined SECT patients**	
Gender (*n*)	
Male	20
Female	22
Age	3.3^a^ (6 months–12 years)
PRISM score	32^a^ (26–39)
PELD score	28^a^ (25–29)
Etiology	(*n*)^b^
Hepatitis-B	8
Hepatitis-C	2
Paracetamol	2
Nonparacetamol drug	10
Toxication (nondrug)	6
Cause unknown	13

ICU, intensive care unit; LTX, liver transplant; PELD, Pediatric End-Stage Liver Disease; PRISM, Pediatric Risk of Mortality; SECT, supportive extracorporeal therapy.

^a^ Median (minimum – maximum).

^b^ Number.

CVVHDF was performed using a renal replacement machine (Fresenius Medical Care, Bad Homburg, Germany) and a hemodiafiltration kit (Multifiltrate Kit midi CVVHDF 400-Pediatric). Continuous 24 h/day citrate-Ca local circuit anticoagulation was performed. The blood flow rate was 3–5 ml/kg/min, the dialysate flow rate was 180–300 ml/kg/h, and the filtration rate after dilution was 36–60 ml/kg/h. The dialysate and replacement solutions used were MultiBic, MultiPLUS, Ci-Ca Dialysate, Ci-Ca Dialysate-K2 PLUS, Ci-Ca Dialysate-K4 PLUS, and 4% sodium citrate. The procedure was continued until LTX or recovery of encephalopathy.

PEX was performed using a continuous renal replacement machine (Fresenius Medical Care, Bad Homburg, Germany) and the Fresenius Plasma Exchange Kit (multiFiltrate Kit MPS P1 DRY-Pediatric). Sessions used an equal volume of FFP replacement without anticoagulant administration by calculating 30 cc/min, 50 cc/kg, and 1.5 volumes. The treatment was applied twice a day for 2–4 h. The PEX circuit was connected as a side flow to the CVVHDF circuit and removed after each treatment session; the CVVHDF continued.

Vital findings were obtained from noninvasive and invasive monitoring recordings in the ICU, and laboratory values were obtained before and immediately after the combined CVVHDF + PEX treatment. PEX treatment with CVVHDF was calculated as hours and the number of treatments.

The retrospective study was approved by the institutional review board of Memorial Sisli Hospital with a date of 03/06/2022 and number 521/22. In addition, these approvals were included in intensive care hospitalization approvals. Patient data were obtained from the hospital information system. This study was carried out at Memorial Sisli Hospital Liver Transplant Intensive Care Unit.

### Statistical analyses

SPSS version 21 (IBM Corp., Armonk, NY, United States) was used for all statistical analyses. Kolmogorov–Smirnoff analysis was used to analyze the normality of the study data. The mean values were used for normally distributed data, and the median (IQR) values were used for non-normally distributed data. A comparison of combined CVVHDF + PEX pre- and post-session laboratory values was performed using the Wilcoxon test. The Mann–Whitney *U* test was used to compare the data of patients with and without LTX. A *P*-value less than 0.05 (*P* < 0.05) was considered statistically significant in this study.

## Results

When the patients were admitted to the ICU, inclusion and exclusion criteria, demographic data, the Pediatric Mortality Risk (PRISM) score, the PELD score, and etiology were recorded (Table 1). All patients included in the study had encephalopathy grade 3 or 4 according to the West Haven classification. The median duration of combined SECT applied to pediatric patients was 8 days. PEX sessions were applied to all patients twice a day. The mean number of sessions was 12.39, with a minimum of 6 and a maximum of 25 sessions. The median duration of CVVHDF was 168.96 h (7.04 days), with a minimum of 74 h and a maximum of 311 h.

The LTX candidates had significantly higher serum total bilirubin values before the first combined SECT than the survivors (recovery ALF). However, alanine transaminase (ALT) and aspartate transaminase (AST) values were significantly lower. The survivors had significantly lower serum ammonia, prothrombin time (PT)/international normalized ratio (INR), and total bilirubin values after combined SECT than the LTX candidates (Table 2). In all pediatric patients (22 liver transplanted and 20 nontransplanted patients), serum liver function test (total bilirubin, ALT, AST), ammonia, and PT/INR levels were significantly lower than the first levels after combined SECT. Hemodynamic parameters (i.e., mean arterial pressure, MAP) also improved significantly (Table 3). The serum liver function test (total bilirubin, ALT, AST) values, ammonia, and PT/INR levels were significantly lower after combined SECT than the first levels in patients with and without LTX. Hemodynamic parameters (i.e., MAP) also improved significantly (Table 4).

**Table 2:** Laboratory values of pediatric patients before the first combined SECT and after the last combined SECT.

	Liver transplant^a^ candidate	Patients recovered (from ALF^a^)	*P*-value
**Before combined SECT**			
AST (U/L)	1660 (1210)	2245 (710)	<0.01
ALT (U/L)	1775 (1142)	2340 (730)	<0.01
Lactate (mmol/L)	5.3 (1.5)	6 (2.8)	0.633
Ammonia (µmol/L)	127.5 (19.75)	127 (23)	0.187
Total bilirubin (mg/dL)	16.6 (9.48)	7.5 (3.2)	<0.01
PT/INR	3.4 (0.55)	3.4 (0.5)	0.274
**After combined SECT**			
AST (U/L)	215 (75.25)	219 (101)	0.197
ALT (U/L)	215 (77.5)	226 (89)	0.06
Lactate (mmol/L)	1.7 (0.15)	1.2 (0.6)	<0.01
Ammonia (µmol/L)	76 (16.75)	56 (10)	<0.01
Total bilirubin (mg/dL)	6.25 (2.68)	2.2 (0.7)	<0.01
PT/INR	1.8 (0.1)	1.3 (0.2)	<0.01

ALF, acute liver failure; SECT, supportive extracorporeal therapy; AST, aspartate transaminase; ALT, alanine transaminase; PT, prothrombin time; INR, international normalized ratio.

^a^ Median (IQR).

**Table 3:** Laboratory values of all patients at admission and after treatment.

	Initial values^a^ pre-SECT	Last values^a^ post-SECT	*P-*value
AST (U/L)	1975 (1030)	199.5 (88.5)	<0.01
ALT (U/L)	1980 (919.5)	207 (75.5)	<0.01
Lactate (mmol/L)	5.35 (1.4)	1.6 (0.45)	<0.01
Ammonia (µmol/L)	127 (23.25)	68 (20.75)	<0.01
Total bilirubin (mg/dL)	13 (10.5)	3.9 (3.68)	<0.01
PT/INR	3.4 (0.5)	1.6 (0.43)	<0.01
pH	7.3 (0.1)	7.4	<0.01
MAP	55 (4.25)	66 (3)	<0.01
Creatinine	1.3 (0.4)	0.5 (0.2)	<0.01
Heartbeat	126 (20)	96 (7)	<0.01
PaO_2_/FIO_2_	285 (21.25)	305 (16.25)	<0.01
Bicarbonate	18.15 (20.5)	22.95 (1)	<0.01

SECT, supportive extracorporeal therapy; AST, aspartate transaminase; ALT, alanine transaminase; PT, prothrombin time; INR, international normalized ratio; MAP, mean arterial pressure.

^a^ Median (IQR).

**Table 4:** LTX candidates (underwent liver TX after the last SECT) and nonliver transplant candidates.

	Initial values^a^ pre-SECT	Last values^a^ post-SECT	*P*-value
**LTX candidates**			
AST (U/L)	1605 (1112)	186 (85.25)	<0.01
ALT (U/L)	1650 (1108)	197 (74.5)	<0.01
Lactate (mmol/L)	5.3 (1.33)	1.7 (0.22)	<0.01
Ammonia (µmol/L)	126.5 (23.25)	76 (16)	<0.01
Total bilirubin (mg/dL)	17.55 (7.75)	5.8 (2.77)	<0.01
PT/INR	3.4 (0.55)	1.75 (0.1)	<0.01
pH	7.3 (0.1)	7.4 (0.1)	<0.01
MAP	56 (6)	67 (2)	<0.01
Creatinine	1.4 (0.3)	0.5 (0.22)	<0.01
Heartbeat	122 (23)	96 (5)	<0.01
PaO_2_/FiO_2_	288.5 (26.25)	305 (16.5)	<0.01
Platelets	45 (19)	61 (18)	<0.01
Bicarbonate	18.15 (2.25)	23.1 (1.52)	<0.01
**Nonliver transplant candidates**			
AST (U/L)	2252 (797)	226 (98.25)	<0.01
ALT (U/L)	2345 (712)	225 (85.25)	<0.01
Lactate (mmol/L)	5.35 (1.58)	1.25 (0.58)	<0.01
Ammonia (µmol/L)	127.5 (23.5)	56 (10.75)	<0.01
Total bilirubin (mg/dL)	7.85 (3.05)	2.25 (0.67)	<0.01
PT/INR	3.4 (0.58)	1.35 (0.28)	<0.01
pH	7.3 (0.1)	7.4 (0.1)	<0.01
MAP	55 (3)	66 (2)	<0.01
Creatinine	1.15 (0.45)	0.5 (0.2)	<0.01
Heartbeat	127 (16)	95 (10)	<0.01
PaO_2_/FiO_2_	280 (22.5)	307 (18.75)	<0.01
Platelets	5 (31)	86 (24)	<0.01
Bicarbonate	18.05 (1.93)	22.8 (0.85)	<0.01

LTX, liver transplantation; SECT, supportive extracorporeal therapy; AST, aspartate transaminase; ALT, alanine transaminase; PT, prothrombin time; INR, international normalized ratio; MAP, mean arterial pressure.

^a^ Median (IQR).

Hypernatremia (five patients) and metabolic acidosis (four patients) developed during PEX in the combined SECT. These improved with hemodiafiltration in SECT. Additionally, four patients had mild skin rash due to FFP used during PEX; the procedure was stopped for these patients. Fresh-frozen plasma in the process was replaced with a new one. Once the skin rashes passed, the procedure continued.

## Discussion and conclusions

We evaluated a group of 22 liver transplanted and 20 nontransplanted pediatric patients who underwent combined SECT and followed up in the LTX ICU. In our study, encephalopathy could not be permanently reduced below grade 3 in 22 patients who underwent LTX; however, it showed a fluctuating course, and there was no increase in the encephalopathy grade or worsening of hepatic coma. This shows that the electrolyte and fluid balance needed to control intracranial pressure (ICP), as reported in the literature, is controlled tightly and precisely. Therefore, combined SECT can be used as a bridge to stabilize candidates for LTX. Encephalopathy in the 18 patients who recovered clinically regressed to grade 1 and below; however, two patients who could not recover had no indication for transplantation or could not find a suitable donor and became ex-patients (dead). The grade of encephalopathy in the two ex-patients could not be permanently reduced below grade 3. A significant improvement was achieved in laboratory (biochemical) values and vital signs in all patients who underwent combined SECT and were followed up in the intensive care unit. However, there was no clinical recovery in patients whose encephalopathy grade could not be permanently decreased. Therefore, we believe that the main factor in determining the prognosis is encephalopathy, which is permanently reduced to grade 1 or lower with combined SECT.

Permanent clinical improvement, including the improvement of encephalopathy as well as a low PT/INR value is observed in surviving patients (recovered patients), and has been achieved with consistently high ammonia clearance, as reported in the literature. We believe that this was due to high functional liver capacity or regeneration of liver tissue (12). In addition, the recovery of patients in the paracetamol-induced ALF group without the need for LTX is consistent with the literature (13).

Many case reports of liver failure describe rapid progression to MOF with the development of systemic infection, brain edema, circulatory failure, abnormal coagulation, and metabolic complications (14). Various clinical studies have shown the ability of CRRT techniques, such as CVVHDF, to reduce inflammation and mediators of inflammation in septic or MOF patients (14, 15). CRRT can be used safely in even the most critical cases associated with circulatory failures, such as cerebral edema (16). If performed continuously, CRRT can effectively remove the densely accumulated substances from the body, not only intravascularly but also extravascularly. Acute kidney injury requiring CRRT is a common manifestation of MOF in ALF; it can develop in 30%–50% of these patients. In addition to removing excess fluid volume from the body, CRRT ensures the excretion of endogenous toxins that cannot be excreted renally. It produces a clinical improvement in patients who are hemodynamically unstable, develop encephalopathy, and go through an extensive inflammation process due to increased and uncleared cytokines. Furthermore, it is widely used to reduce the risk of increased ICP and cerebral edema in patients with severe hyperammonemia (17).

Plasma exchange clears circulating inflammatory cytokines, endotoxins, and neurotoxins formed in patients with ALF. It lowers bilirubin and ammonia levels, corrects coagulopathy, and regulates the antimicrobial response, thereby reducing transplants, with hemodynamic improvement (17, 18). These reduced substances have been reported to be important mediators in developing both HE and MOF in ALF. Mao et al. showed that bilirubin, coagulopathy, and ammonia levels were corrected with PEX treatment (19, 20). Combined SECT is not a new practice; simultaneous dialysis and PEX were first introduced in 1999 and these have been reported in the literature as serial or parallel connected tandem procedures (21). According to Matsubara et al. an artificial liver support system, such as continuous hemofiltration with PEX, can effectively remove medium-molecular-weight substances and is thus effective in treating hepatic coma (22–24). Yoshiba et al. reported that combining PEX and hemodiafiltration could improve hepatic coma in many patients; using this artificial liver support system, the survival rate in 67 patients with fulminant hepatitis was 56% (25). Compared with previous combined SECT reports, the transplant-free survival was similar. On the other hand, although the causative etiology is different, in 38% of pediatric ALF patients who do not undergo liver TX, Kathemann et al. reported a 59% mortality rate in those who did not undergo SECT and were left to recover spontaneously (26). These patients should be supported in the ICU until their liver function is restored or LTX is performed, with various types of monitoring and measures, including intensive treatment.

We believe that our multidisciplinary approach to LTX, performed at the proper time, is effective in preventing mortality and ensuring neurological stability. We attributed the low mortality rate in our study to the use of early and aggressive artificial liver support.

Many studies have shown that rapid, large-volume FFP administration can produce side effects such as hypernatremia, metabolic alkalosis, and colloid osmotic pressure (COP) during the PEX procedure. Furthermore, it has been reported that large-volume FFP administration can lead to complications, such as brain and pulmonary edema (27). The first randomized control trial (RCT) describing the benefits of the PEX procedure in patients with ALF was conducted in 2016 by Larsen et al. (28). Persistent cerebral edema and hepatic coma in patients with ALF are important because they show the abrupt development of irreversible cerebral damage. This situation was also reported by Bernal and Wendon as a contraindication for LTX (29). Since 2007, the combined use of PEX and CVVHDF has been ongoing in our unit. By using this method, we efficiently remove hepatic toxins and minimize the side effects of PEX administration alone, such as hypernatremia, COP, and metabolic alkalosis (30, 31).

Nevertheless, there is a need for randomized controlled studies on this subject since our study is single-center and retrospective and there is no control group.

Combined CVVHDF and PEX supportive treatment significantly improved the biochemical parameters and clinical findings, including encephalopathy. Furthermore, it was found to have a major effect on patient survival, with 4.76% mortality and 42.8% transplant-free survival. Therefore, we believe that this combined therapy can be used as supportive therapy for recovery or bridging purposes in patients with ALF or candidates for LTX while waiting for liver regeneration or a suitable donor.

## Data Availability

The original contributions presented in the study are included in the article/Supplementary Material, further inquiries can be directed to the corresponding author.
